# Iron-tracking strategies: Chaperones capture iron in the cytosolic labile iron pool

**DOI:** 10.3389/fmolb.2023.1127690

**Published:** 2023-02-02

**Authors:** Caroline C. Philpott, Olga Protchenko, Yubo Wang, Lorena Novoa-Aponte, Andres Leon-Torres, Samantha Grounds, Amber J. Tietgens

**Affiliations:** Genetics and Metabolism Section, Liver Diseases Branch, National Institutes of Diabetes and Digestive and Kidney Diseases, National Institutes of Health, Bethesda, MD, United States

**Keywords:** poly C binding protein1, PCBP1, ferroptosis, ferroportin, enteroid, ferritin

## Abstract

Cells express hundreds of iron-dependent enzymes that rely on the iron cofactors heme, iron-sulfur clusters, and mono-or di-nuclear iron centers for activity. Cells require systems for both the assembly and the distribution of iron cofactors to their cognate enzymes. Proteins involved in the binding and trafficking of iron ions in the cytosol, called cytosolic iron chaperones, have been identified and characterized in mammalian cells. The first identified iron chaperone, poly C-binding protein 1 (PCBP1), has also been studied in mice using genetic models of conditional deletion in tissues specialized for iron handling. Studies of iron trafficking in mouse tissues have necessitated the development of new approaches, which have revealed new roles for PCBP1 in the management of cytosolic iron. These approaches can be applied to investigate use of other nutrient metals in mammals.

## Introduction

Nearly all organisms require iron to form the cofactors that activate essential enzymes driving cellular metabolism, small molecule synthesis, and organism self-renewal. In mammals, iron is the most abundant metal. The iron-containing heme cofactors found in oxygen-carrying proteins of red blood cells (hemoglobin) and muscle (myoglobin) are the most abundant metal-containing proteins in mammals (Dreyfuss et al., 1988). Iron deficiency is a clinical disorder that usually manifests as microcytic anemia with low levels of hemoglobin in blood. However, the essential requirement for iron cofactors extends beyond heme and the oxygen-carrying capacity of red blood cells.

Proteins coordinate iron cofactors in the form of heme, iron-sulfur clusters and mono- and di-nuclear iron centers. Heme is synthesized in every cell *via* an 8-step pathway that begins in the mitochondria, shifts to the cytosol, and ends in the mitochondrial inner membrane with the insertion of iron into protoporphyrin IX (Aasheim et al., 1994). Heme is poorly soluble in aqueous environments and has peroxidase activity that can cause oxidative damage. Nevertheless, heme enzymes locate to compartments throughout the cell; thus, heme needs to be safely transported across membranes and escorted through the cytosol to be inserted into its cognate enzymes.

Iron-sulfur clusters are assembled primarily in the mitochondrial matrix where they are inserted as [2Fe-2S] or [4Fe-4S] clusters into recipient apoenzymes of the mitochondria (Shi et al., 2008). An undefined sulfur-containing molecule is exported from the mitochondria *via* the transporter Atm1 (ABCB7) for use in cytosolic iron-sulfur cluster assembly. Although this exported compound has not been clearly identified, some studies propose that a [2Fe-2S] cluster coordinated by glutathione may be transported (Nandal et al., 2011; Leidgens et al., 2013). A parallel system of assembly factors is proposed to operate in the cytosol (Frey et al., 2014). The earliest steps in cytosolic Fe-S assembly are not well-defined, but both [2Fe-2S] and [4Fe-4S] clusters are required in the cytosol. Multiple scaffold/assembly proteins coordinate the [4Fe-4S] clusters and are required for their subsequent transfer to cytosolic and nuclear targeting factors (Frey et al., 2016). Iron-sulfur clusters can be sensitive to both oxidation and reduction and are not stable as uncoordinated, “free” molecules in solution. Therefore, both heme and iron-sulfur clusters require distribution systems that facilitate their transfer from biosynthetic/assembly machineries to recipient apoenzymes. Protein chaperones for heme and iron-sulfur clusters have been identified and the molecular details of their distribution systems, while incomplete, are gradually coming into focus. Simple mono-nuclear and dinuclear iron centers that activate the “non-heme” iron enzymes consist of Fe(II) and do not require dedicated assembly systems. They do require distribution systems, however, and recent studies reveal the roles that the iron chaperones play in maintaining cellular iron balance and utilization (Patel et al., 2019).

Iron ions are taken up by cells and initially deposited into the cytosol by iron-specific transporters, where they may be routed to different cellular destinations and used for various purposes (Patel et al., 2019). Most of the iron is used in mitochondria to synthesize heme and Fe-S clusters, to metallate mitochondrial non-heme iron enzymes, and, in some cells, to be stored in mitochondrial ferritin. Cytosolic iron is also used to form Fe-S clusters, to metalate non-heme iron enzymes, or oxidized for storage in cytosolic ferritin. Although these iron pools are often discussed as separate entities, they are highly dynamic and exchangeable. Cells exhibit a continuous, basal level of mitochondrial turnover and ferritin degradation in lysosomes, with recovered iron reentering the cytosol. Iron cofactor-containing enzymes have a limited half-life and are also continuously turned over and the iron recycled. Iron cofactors themselves may be damaged or lost, requiring repair or replacement, although less is known about these non-degradative processes. Finally cytosolic iron may be exported *via* the iron efflux pump, ferroportin. Ferritin may also be exported *via* exosomes or non-classical secretion, but the amount of iron exported as ferritin is low (Leffers et al., 1995). This highly dynamic pool of cytosolic iron has been referred to as the “labile iron pool” or LIP and is both kinetically exchangeable and chemically reactive. Theoretically, the cytosolic LIP is coordinated by a complex buffer consisting of large and small molecules. Empirical data indicate the major protein coordinating the LIP in the cytosol is poly C-binding protein 1 (PCBP1).

### PCBP1: The primary cytosolic iron chaperone in mammalian cells

**FIGURE 1:**
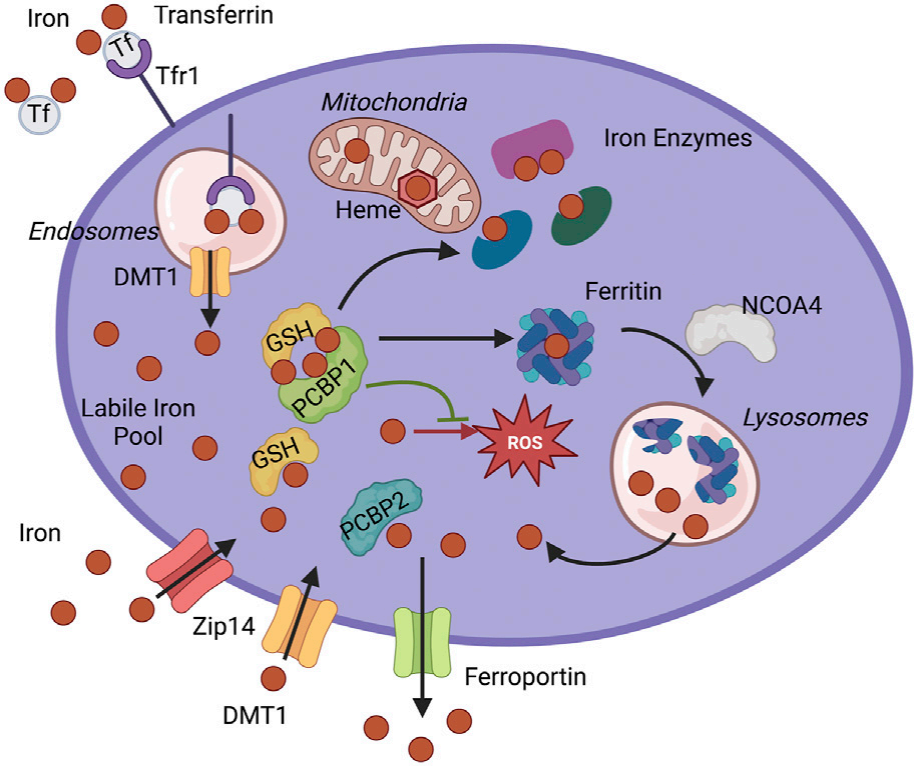
Iron chaperone management of intracellular iron. PCBP1 and PCBP2 ensure the safe distribution of iron into three main pools of cytosolic iron: storage, use in iron enzymes, and labile iron pool (LIP). Iron enters cytosol as non-transferrin-bound iron through Divalent metal transporter 1 (DMT1) or Zip14 and as transferrin-bound iron through Transferrin receptor 1 (Tfr1). The iron-transferrin complex dissociates in the acidic lumen of the endocytic compartment, and iron is transported into the cytosol by DMT1. Iron enters the cytosolic labile iron pool (LIP) as Fe(II) and is primarily coordinated by reduced GSH and iron chaperones, PCBPs. PCBP1 and possibly PCBP2 deliver iron for storage in ferritin. Sequestered iron is freed after targeting ferritin for degradation by NCOA4. Iron goes to the different cellular compartments, mitochondria, and cytosol to synthesize heme and Fe-S cofactors or other iron proteins. Iron chaperones ensure proper and efficient metalation of mono-and di-iron enzymes, such as iron proteins prolyl hydroxylase (PHD2) and deoxyhypusine hydroxylase (DOHH). Iron chaperones in the complex with GSH also safeguard labile iron pools to protect proteins and cellular compartments, like mitochondria, from harmful reactive oxygen species (ROS) or control efflux through iron exporter ferroportin.

PCBP1 was initially identified as an RNA-binding protein found in heterogeneous nuclear ribonucleoprotein complexes and is also known as hnRNP E1 (Dreyfuss et al., 1988) (Aasheim et al., 1994). Decades later, PCBP1 was identified in a yeast genetic screen for human proteins that bind iron and deliver it to ferritin in cells (Shi et al., 2008) (Leidgens et al., 2013). Subsequent studies conducted in human cells indicated that PCBP1 is not only an iron chaperone for ferritin, but it also has the capacity to bind cytosolic Fe (II) and deliver it directly to other apoenzymes with iron centers. These enzymes include those with mononuclear iron centers, such as the prolyl hydroxylases that regulate hypoxia-inducible factors (Nandal et al., 2011) or dinuclear iron centers, such as deoxyhypusine hydroxylase (Frey et al., 2014). Mechanistically, metalation of these non-heme iron enzymes occurs through associative transfer of the Fe (II) cofactor. Associative transfer implies that direct contact between the iron ion on the chaperone and the iron-coordinating side chains of the recipient apoenzyme should occur to facilitate ligand exchange. A model of the pools of iron managed by chaperones in the cell is depicted in Figure 1. Proteomic studies of the PCBP1 interactome revealed that a component of a cytosolic [2Fe-2S] chaperone complex, BolA2, formed an iron-coordinating, cochaperone complex with PCBP1. This PCBP1-Fe(II)-BolA2 complex was required for the assembly of [2Fe-2S] complexes on BolA2-Glrx3 and links the cytosolic LIP to the cytosolic Fe-S cluster assembly system (Frey et al., 2016; Patel et al., 2019).

A combination of cell-based and *in vitro* approaches has revealed molecular details about the coordination of iron on PCBP1. PCBPs share similar structures that consist of three highly-conserved, nucleic acid-binding modules, called hnRNP K-homology (KH) domains (Leffers et al., 1995). Each KH domain is separated by variable linker domains with lower sequence conservation. The KH domains of PCBP1 and PCBP2 have been structurally characterized bound to cytosine (C)-rich oligonucleotides (Du et al., 2007; Yoga et al., 2012). In PCBP1 and PCBP2, KH domains 1 and 2 form a dimerized domain that binds with high affinity a single oligonucleotide that contains a tandem poly-C motif. The KH3 domain of PCBPs also has an intermediate affinity for both C-rich RNA and DNA oligonucleotides. Initial characterization of iron-binding sites on PCBP1 indicated a 3 Fe:1 protomer stoichiometry with a single high-affinity site and two intermediate-affinity sites (Shi et al., 2008), suggesting that each of the KH domains could also be an iron-binding module. More recent studies characterized the iron-binding site on the KH3 domain of PCBP1. Using site-directed mutagenesis and radiolabeled iron pull-downs in cells, studies identified conserved cysteine and glutamate residues required for Fe(II) binding. Furthermore, these studies found that a molecule of non-covalently bound glutathione was also required for iron coordination in cells. Amino acid residues independently required for glutathione binding on KH3 were also identified. These data indicated that iron-glutathione complexes were the relevant species for PCBP1 iron coordination and added further evidence that glutathione is the main small-molecule ligand for iron in the LIP (Patel et al., 2019). Subsequent *in vitro* studies using purified, recombinant KH3 confirmed the high-affinity binding of an Fe(II)-glutathione complex and the identity of putative iron- and glutathione-binding residues.

The iron- and nucleic acid-binding sites on the KH3 domain were potentially conserved on the KH1 and KH2 domains. Furthermore, because these binding sites are located on opposite faces of the KH domains, nucleic acid-binding and iron-binding activities were potentially separable. In a recent study, substitution mutations of putative iron-binding residues on each KH domain successfully produced a variant of PCBP1 that completely lacked iron-binding activity, while completely preserving oligonucleotide-binding activity (Patel et al., 2021). Similarly, an analogous variant with mutations in nucleic acid-binding residues lost DNA binding without affecting iron binding. Expression of these variants in cells depleted of endogenous PCBP1 enabled the assignment of various PCBP1-depletion phenotypes to specific binding activities of PCBP1. Cell cycle delays and DNA damage phenotypes were attributable to loss of iron-binding activity while loss of viability was attributed to loss of nucleic acid binding. Similar results were observed in a mouse model of hepatocyte-specific PCBP1 deletion with AAV-mediated transduction of PCBP1 variants. Further studies will determine the relative contributions of these activities to the functions of PCBPs.

#### PCBP2: A secondary iron chaperone with specialized activities

Although the initial genetic screen that produced PCBP1 as an iron chaperone did not identify other PCBP family members as potential iron chaperones, subsequent examination of the paralogous PCBP2, PCBP3, and PCBP4 suggested that they could also potentially have iron chaperone activity (Leidgens et al., 2013). PCBP1 and PCBP2 are 83% identical at the amino acid level and *PCBP1*, an intron-less gene, is proposed to have arisen from a retrotransposed copy of a minor splice variant of PCBP2 (Makeyev et al., 1999). Both PCBP1 and PCBP2 are ubiquitously expressed at high levels in mammalian tissues, a feature necessary for iron chaperone activity. Cell-based studies have suggested that PCBP2 has a lower level of iron chaperone activity for the metalation of ferritin and non-heme iron enzymes (Nandal et al., 2011; Frey et al., 2014; Ryu et al., 2017), but that it also has specific functions as a cytosolic iron chaperone. PCBP2 was shown to specifically interact with the iron transporters divalent metal transporter 1 (DMT1) and ferroportin, facilitating their iron import and efflux activities, respectively (Yanatori et al., 2014; Yanatori et al., 2016). Further studies indicated that PCBP2 can also complex with heme oxygenase to capture iron released during heme degradation (Yanatori et al., 2017).

## Animal models of conditional PCBP1 deletion reveal new roles for iron chaperones

Studies of human physiology make clear that cells and tissues may have vastly different systems and requirements for handling and using iron. These differing tissue-specific patterns of iron homeostasis must also be integrated so that total body iron remains in balance, without overload or deficiency. Some cells and tissues are thought of as “professional” iron-handlers and exhibit high levels of iron transport, iron storage or iron utilization. Examination of the roles of PCBP iron chaperones in professional iron handling tissues has revealed molecular details of the functions of iron chaperones. Global deletion of the genes encoding PCBP1 or PCBP2 in the mouse results in lethality in early embryogenesis (Ghanem et al., 2016; Ryu et al., 2017). Tissue-specific, conditional deletion models have proven to be much more informative. Here, we describe approaches used within our laboratory to understand the roles of iron chaperones in cells and tissues.

## Ferritin iron storage in terminal red blood cell differentiation

Because developing erythrocytes require a tremendous uptake of iron to initiate and complete the heme synthesis needed for terminal differentiation, we hypothesized they were also likely to require iron chaperone activity. In a mouse model of inducible PCBP1 deletion, mice developed microcytic anemia that was traced to a defect in PCBP1-mediated ferritin iron storage (Ryu et al., 2017). *In vitro* and *ex vivo* murine models of proerythroblast development demonstrated that the earliest phases of terminal differentiation involved PCBP1-dependent storage of iron in ferritin. In PCBP1-depleted cells, this ferritin iron accumulation was impaired and associated with diminished heme and hemoglobin synthesis, which in the animal led to a microcytic anemia typical of iron deficiency. These studies relied on a new approach to studying erythroid development: an inducible erythroid differentiation system and the tracking of intracellular iron using radiotracers.

We used an estradiol-inducible murine cell line, G1E-ER4, that recapitulates terminal differentiation from the proerythroblast to orthochromatic erythroblast stage (Welch et al., 2004). The late stages of red blood cell development feature tremendous uptake of transferrin-bound iron, which is initially stored in ferritin and is subsequently transferred to mitochondria for insertion into heme. Heme is then incorporated into nascent globin chains to form hemoglobin. Radioisotopes of iron have long been used to measure uptake and absorption of iron in cells and animals. By labeling the developing erythroid cells with ^55^Fe, we were able to track the flux of iron through 48 h of development: first in ferritin, then in heme, and finally in hemoglobin. We traced ^55^Fe in native gels, which separate ferritin iron from hemoglobin iron, and measured the iron by phosphorimaging. ^55^Fe in newly synthesized heme was organically extracted and measured by scintillation counting. Using RNA interference-mediated depletion of PCBP1, we measured the impaired delivery of ^55^Fe to ferritin, the reduced synthesis of heme, and lowered levels of hemoglobin. Conversely, G1E-ER4 cells depleted of PCBP2 exhibited higher levels of ferritin storage and heme and hemoglobin synthesis, suggesting a competition between PCBP1 and PCBP2 for cytosolic iron. We then shifted to our mouse model of PCBP1 depletion, which relies on a tamoxifen-inducible Cre-*loxP* system with *PCBP1* flanked by *loxP* sites and a transgene carrying the *Cre* recombinase-estrogen receptor (Cre-ER^T2^) fusion protein (Ryu et al., 2017). Bone marrow from tamoxifen-treated animals exhibited lower levels of PCBP1 and lower levels of heme while peripheral blood analysis showed microcytic anemia. Finally, we isolated proerythroblasts from spleens of phenylhydrazine-treated animals lacking PCBP1 and traced their trafficking of ^55^Fe through *ex vivo* differentiation. The impaired ferritin iron storage and subsequent poor hemoglobin synthesis initially observed in G1E-ER4 cells was again observed in the differentiating splenic proerythroblasts. By combining radiotracer studies with genetic models of PCBP1 depletion, we uncovered the critical role of PCBP1-mediated iron storage in ferritin as an important early step in red blood cell terminal differentiation.

## Capture of dietary iron in intestinal epithelium

Human adults require 1–2 mg of iron per day to compensate for the daily loss of iron (Leong et al., 2012; McKie et al., 2012). This requirement is met with dietary iron, which is primarily absorbed from the duodenum and proximal jejunum (Collins et al., 2018). Unlike other metals, iron cannot be excreted in humans. Thus, dietary iron absorption from the small intestine must be strictly regulated to ensure sufficient uptake to meet body iron requirements while simultaneously preventing oxidative damage to cells and tissues by excess iron accumulation. Both insufficient and excess iron absorption lead to a host of human health problems, such as iron deficiency anemia and hereditary hemochromatosis, respectively (Pasricha et al., 2021).

**FIGURE 2:**
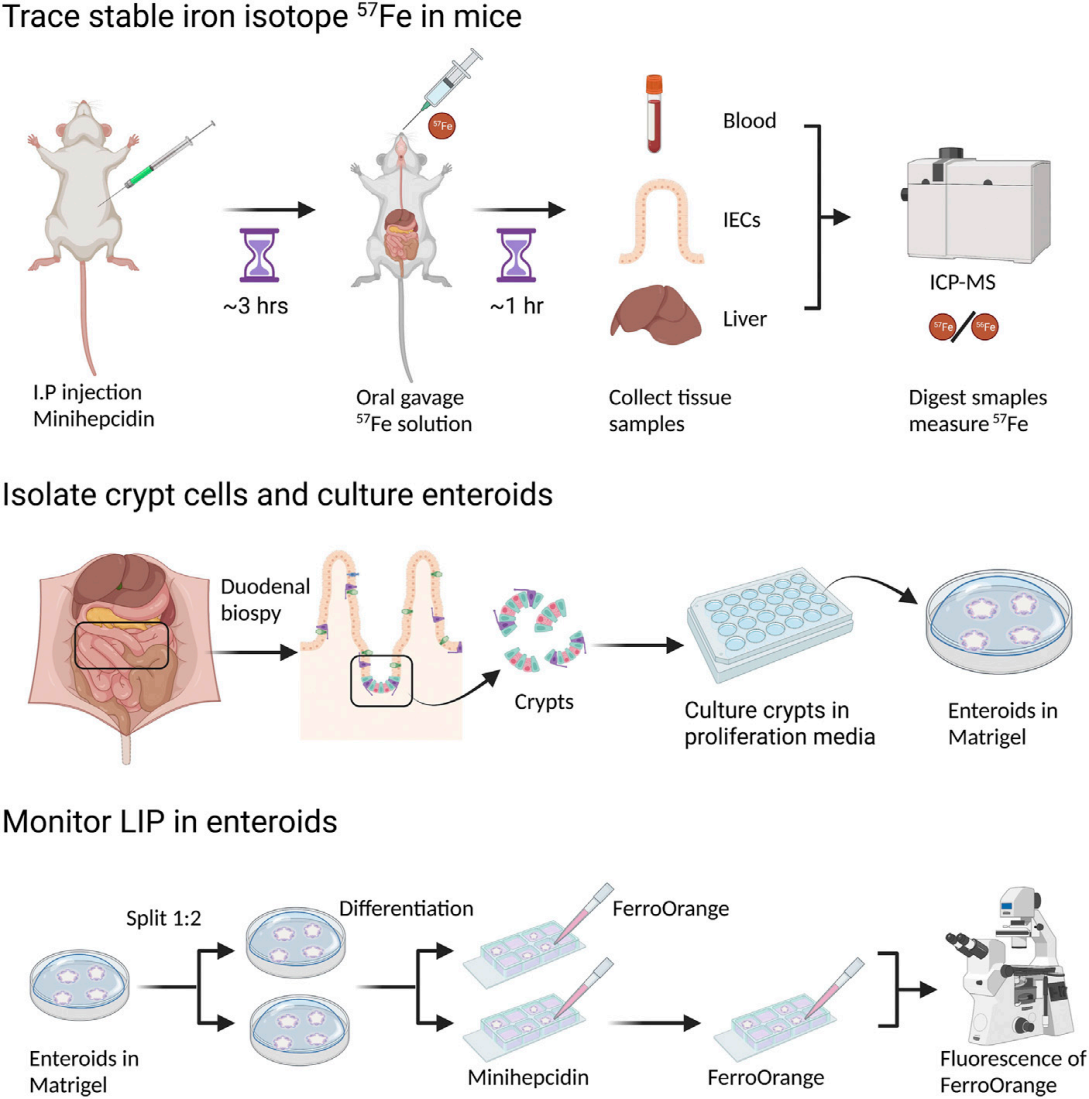
Measurement of dietary iron absorption using stable iron isotope ^57^Fe. ^57^FeSO_4_ solution is administrated through oral gavage to mice that are fasted overnight. One hour after ^57^Fe administration, mice are sacrificed and tissue samples, such as duodenal epithelial cells and liver, are collected. For ICP-MS analysis, samples need to be digested in concentrated HNO_3_. ^57^Fe content in different tissues can be determined by ICP-MS though the difference of ^57^Fe/^56^Fe ratio before and after ^57^Fe administration. To block dietary iron absorption through duodenal ferroportin, soluble minihepcidin is injected intraperitoneally into mice several hours before ^57^Fe administration.

Dietary iron occurs in two forms, heme and non-heme iron. Studies in humans suggest that, while heme iron accounts for only about 10% of total dietary iron (Leong et al., 2012), it can comprise up to 40% of the total iron absorbed due to its bioavailability (Hurrell and Egli, 2010). However, the mechanism of heme iron absorption in animals is not well understood (Gulec et al., 2014) and in the mouse, nearly all dietary iron absorption occurs through non-heme iron (Dutt et al., 2022). Duodenal epithelial cells take up dietary iron through DMT1 at their apical membranes (Gunshin et al., 1997; Romero et al., 1997; Yanatori and Kishi, 2019). After dietary iron is imported into the enterocyte by DMT1, intracellular iron may be stored in ferritin, the iron storage protein, or be exported to the systemic circulation *via* the only iron exporter, ferroportin, which is located in basolateral membranes (Abboud and Haile, 2000; Donovan et al., 2000; McKie et al., 2000; Rishi et al., 2021). At the systemic level, intestinal iron absorption is regulated by hepcidin, a 25-amino acid-peptide hormone secreted by the liver and other tissues that binds ferroportin on basolateral membranes of intestinal epithelial cells and blocks its activity (Drakesmith et al., 2015; Nemeth and Ganz, 2021). If iron stored in enterocytes is not absorbed, it will be lost when the senescent epithelial cells are shed into the gut lumen. Thus, controlling iron efflux through ferroportin is the key regulatory step in dietary iron absorption. However, several studies have shown that ferroportin is not always responsive to the regulation of hepcidin (Frazer et al., 2017; Hanudel et al., 2022). Previous studies show that both PCBP1 and PCBP2 deliver iron to ferritin (Shi et al., 2008; Leidgens et al., 2013). In addition, PCBP2 has been shown to associate with DMT1 and ferroportin in yeast and mammalian cells (Yanatori et al., 2014; Yanatori et al., 2016). However, whether the iron chaperone activities of PCBP1 and PCBP2 play an important role in iron absorption through the small intestine or in maintenance of systemic iron balance has not been explored. There are still many questions that need to be answered before we can fully understand the mechanism of iron absorption through small intestine and intracellular iron trafficking in intestinal epithelial cells. Here we present some new approaches that became popular in the past decade and will be useful for future studies of iron absorption (Figure 2).

Radioactive isotopes ^55^Fe and ^59^Fe were widely used in iron absorption studies *in vivo*, both to determine mucosal iron uptake and to trace iron absorption from the small intestine to other organs. However, both ^55^Fe and ^59^Fe emit radioactive particles, which requires special laboratory equipment and skills, as well as specific handling and disposal of animals and samples treated with radioisotopes. While ^55^Fe was initially used in human studies, the higher energy of ^59^Fe rendered it inappropriate for human use. Stable isotopes of iron are becoming the preferred form of iron for biological and biophysical studies. There are four natural iron isotopes--^54^Fe, ^56^Fe, ^57^Fe and ^58^Fe, and their natural isotopic abundance ratios are 0.058, 0.917, 0.021 and 0.002, respectively (Taylor et al., 1992; Meija et al., 2016). The stable iron isotopes, ^57^Fe and ^58^Fe, are more commonly used for iron absorption studies in both human (Chen et al., 2005; Stoffel et al., 2017; Speich et al., 2021) and mouse (Fiorito et al., 2012; Nai et al., 2021) because of their low natural abundance. Historically, stable isotopes were not widely used in iron absorption studies due to their high cost and the limited availability of thermal ionization mass spectrometry (TIMS) for isotope ratio analysis (Chen et al., 2005). However, use of stable isotopes has increased with the development of high-resolution inductively-coupled plasma mass spectrometry (ICP-MS), which allows lower costs, easier sample preparation, and greater accessibility of isotope ratio analysis. ^54^Fe, ^57^Fe and ^58^Fe allow studies on long-term and short-term iron absorption through direct measurement of these isotopic iron tracers from blood and other tissues. Because there are very little safety concerns, these studies using stable iron isotopes could cover various groups of study subjects, such as young children, adults, and pregnant women (Moretti et al., 2015; Cai et al., 2018; Delaney et al., 2020). As humans and mice share the key iron transporters and iron regulation mechanisms, many studies on iron absorption and iron homeostasis are carried out in mice, and more mouse studies use stable iron isotopes to trace iron trafficking and iron absorption (Fiorito et al., 2012; Chakrabarti et al., 2015; Sangkhae et al., 2020).

Tracer studies of iron absorption have become more powerful with the use of both genetic and pharmacologic methods to perturb iron trafficking. Minihepcidin is a synthetic peptide which contains the 9N-terminal amino acids of human hepcidin (Preza et al., 2011; Ramos et al., 2012). Based on mouse studies, minihepcidin can mimic the biological function of natural hepcidin and block iron export through ferroportin, making it a potential drug candidate for treatment or prevention of iron overload. Minihepcidin has been used in murine studies on iron absorption and iron homeostasis (Guggisberg et al., 2022; Hanudel et al., 2022) and can be used in conjunction with tracer studies to produce rapid and complete inhibition of ferroportin-mediated iron efflux.

*In vitro* studies of intestinal transport have relied in the past on immortalized cell lines cultured on membranes to mimic the epithelial layer of the intestine. Enteroids (intestinal organoids) are three-dimensional epithelial structures grown *ex vivo* from isolated intestinal stem cells (Sato et al., 2009). Human and mouse enteroid models are much more physiologically similar to mammalian intestine *in vivo* when compared to widely-used cultured cell models, such as Caco-2 and HT29 cells (Zachos et al., 2016; Rodrigues and Failla, 2021). Stem cells in undifferentiated enteroids can proliferate for many passages, allowing their propagation in culture. Under differentiation conditions, the stem cells can differentiate into all other cell lineages of intestinal epithelium, such as enterocytes, goblet cells, and enteroendocrine cells. Because of their capacity to recapitulate the major structural and functional characteristics of epithelium *in vivo,* enteroids are widely used in studies of cancer, gastrointestinal infection, and drug development (Zou et al., 2019; Schwartz et al., 2021). Enteroids were also used as models of small intestinal ion and dietary nutrient transport (Foulke-Abel et al., 2016; Pierson et al., 2018). The use of minihepcidin in enteroid models can recapitulate the systemic regulation of iron efflux by hepcidin *in vivo*. Compared to animal models, enteroids represent an environment that is both stable and readily probed with drugs and small molecules. Simply through switching on and off the systemic iron regulator, the enteroid model provides a powerful tool to study iron uptake and efflux, as well as intracellular iron trafficking through intestinal epithelial cells.

**FIGURE 3:**
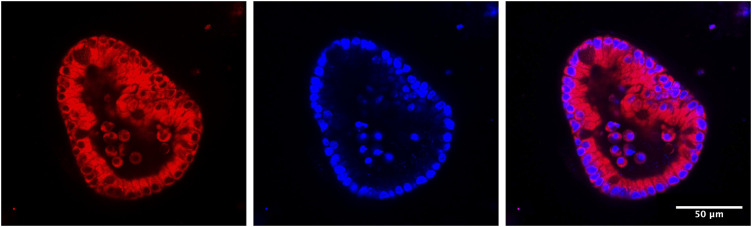
Establishment of mouse 3-D enteroid culture to monitor LIP in live enteroids. 1–2 cm of mouse duodenum is isolated, washed and cut into small pieces. Crypt cells are separated from other tissue fragments through EDTA incubation and centrifugation. Next, crypt cells are mixed with Matrigel and then plated on a 24-well plate. In the following days, the open crypts will close to form spherical enteroids. Stem cells in enteroids will keep proliferating when they are in enteroid culture media (with Wnt and other growth factors). If growing in differentiation media (without Wnt in the media), stem cells will differentiate into different types of intestinal cells, such as enterocytes, goblet cells, etc. To observe LIP in living enteroids under different conditions, e.g., normal condition or ferroportin is blocked by minihepcidin, enteroids are isolated from Matrigel and treated with FerroOrange, a fluorescent probe for labile iron. After half an hour, LIP can be observed under fluorescent confocal microscopy.

Although enteroid cultures are typically conducted on a scale too small for isotopic tracer studies, they are amenable to microscopic studies of iron trafficking. Physiologically, dietary iron that is taken up by enterocytes enters the cytosolic LIP. There, iron can be retained in the cytosol, transferred to ferritin for storage, or exported *via* ferroportin. Our previous studies indicate that PCBPs may serve as buffers for iron in the cytosolic LIP and control its trafficking. To track the LIP in enteroids, we have used recently developed fluorescent iron indicators. RhoNox-4, commercially available as FerroOrange, is a highly-sensitive fluorescent probe for intracellular labile iron based on an Fe(II)-mediated deoxygenation of a tertiary amine N-oxide (Hirayama, 2019; Hirayama et al., 2020). Compared to conventional fluorescent probes of Fe(II), such as calcein and PhenGreen-SK, RhoNox-4 selectively responds to Fe(II) with a direct fluorescent signal and without interference by other metals in living cells. The sensitivity of RhoNox-4 suggests that it may detect both “free” Fe(II) and iron bound by PCBP1, based on its kinetic lability and the dissociation constant (K_d_) of PCBP1-bound iron. Use of this fluorescent iron indicator will help us to understand the roles of PCBPs as iron chaperones in iron absorption through measurement of the LIP in living enterocytes of enteroids (Figure 3).

## Detoxification of iron in liver

The role of iron chaperones is especially crucial for liver function because the liver is the central iron storage organ in the body. The portal circulation directly connects the liver to the flow of dietary iron from the gut and the secretion of recycled red cell iron from splenic red pulp macrophages. Because liver cells are exposed to significant fluctuations of plasma iron, proper handling of the intracellular labile iron pool is critical for liver function and systemic iron homeostasis. Liver diseases such as non-alcoholic fatty liver disease (NAFLD), non-alcoholic steatohepatitis (NASH), and alcoholic liver disease (ALD) are associated with impaired iron regulation (Nelson et al., 2012; Britton et al., 2016; Ali et al., 2022). Animal studies in mice and rats also demonstrate that excess iron leads to oxidative stress, steatosis, and liver damage (Handa et al., 2016; Folgueras et al., 2018). We examined the role of the PCBP1 iron chaperone in the liver. We bred PCBP1 flox/flox mice carrying the Alb-Cre transgene, which mediates hepatocyte- and cholangiocyte-specific Cre recombinase expression. The hepatic loss of PCBP1 resulted in liver damage with steatosis (elevated liver triglycerides) and dying hepatocytes (elevated plasma alanine transaminase, ALT) (Protchenko et al., 2021). Several observations indicate that loss of iron chaperone results in liver iron toxicity. First, although PCBP1-depleted livers are iron-deficient, dietary iron supplementation did not improve liver parameters. In contrast, a low-iron diet prevented the development of steatosis. Second, iron-overloaded livers and PCBP1-deleted livers share similar patterns in their transcriptomes and lipidomes. Iron overload and PCBP1 deletion increased the expression of lipid biosynthesis genes and activated transcriptional pathways for the Nrf2-mediated oxidative stress response, ferroptosis signaling, and DNA damage response. Also, iron-overloaded and PCBP1-deficient livers produced similar profiles of oxidized lipid species, indicating intracellular iron toxicity.

In the lipid-rich environment of hepatic tissue, the accelerated lipid peroxidation due to the loss of the iron chaperone can affect many aspects of cellular metabolism. PCBP1-deficient livers exhibited depletion of mitochondrial lipids cardiolipin and coenzyme Q. These lipid changes contributed to mitochondrial damage and manifested in multiple signs of mitochondrial dysfunction in PCBP1-depleted livers (Jadhav et al., 2021). Diets supplemented with antioxidants vitamin E or coenzyme Q partially prevented the development of steatosis and liver damage, and coenzyme Q was required to prevent mitochondrial dysfunction.

*In vitro* visualization of changes in lipid oxidation and labile iron in primary cells from PCBP1-deficient livers is a powerful approach to real-time monitoring of the function of the iron chaperone and intracellular redox status. BODIPY C11 is a lipophilic, ratiometric fluorescent sensor used to quantify lipid peroxidation (Pap et al., 1999). Visualization of intracellular redox status in primary hepatocytes isolated from wild-type and PCBP1-deficient livers using BODIPY C11 confirmed accumulation of oxidized lipids upon loss of the iron chaperone. Hepatocytes deficient in PCBP1 have alterations in the labile iron pool which can be visualized using the fluorescent iron sensor FIP-1. FIP-1 reacts with kinetically-labile, chemically-reactive ferrous iron in cells (Aron et al., 2016). Intact FIP-1 has two FRET-producing fluorophores (fluorescein and Cy3) attached to a Fe(II)-cleavable endoperoxide bridge. Fe(II)-triggered cleavage of the linker separates fluorophores and eliminates FRET. Fluorescence of cleaved and non-cleaved forms of FIP-1 permits ratiometric measurement of labile iron. More iron-reactive fluorescence of FIP-1 was observed in hepatocytes isolated from PCBP1-deleted or iron-loaded wild-type livers. Even as PCBP1-deleted livers were iron-deficient, loss of the iron chaperone increased the chemical reactivity of intracellular iron (II). These studies confirmed the function of PCBP1 as an essential chaperone for iron to counteract the chemical reactivity of Fe(II) and prevent the formation of harmful reactive oxygen species.

The complex phenotype of PCBP1-depleted livers indicates that PCBP1 is integral to ferroptosis defense. Ferroptosis is a non-apoptotic, regulated cell death caused by iron-dependent lipid peroxidation (Stockwell, 2022). Ferroptosis manifestations vary in different cell lines and tissues but share common features, such as elevated levels of iron-mediated lipid peroxidation causing loss of membrane integrity, enhanced cell permeability, swollen mitochondria, and cell death (Stockwell et al., 2017; Jiang et al., 2021). Ferroptosis inducers include drugs that deplete intracellular glutathione or inhibit Gpx4, a glutathione peroxidase that converts lipid hydroperoxides to lipid alcohols (Forcina and Dixon, 2019). Ferroptosis inhibitors are mainly lipophilic antioxidants, such as vitamin E, liproxstatin-1 and coenzyme Q, or iron chelators, such as desferrioxamine. Glutathione is a critical co-factor for Gpx4 activity and also functions to coordinate intracellular iron (Hider et al., 2021). The complex of PCBP1 with iron and glutathione has an essential and yet unexplored role in ferroptosis defense to safeguard reactive, labile iron of the cytosolic LIP.

## The combination of iron chaperone and nucleic acid-binding activities impact PCBP1 functions

**FIGURE 4:**
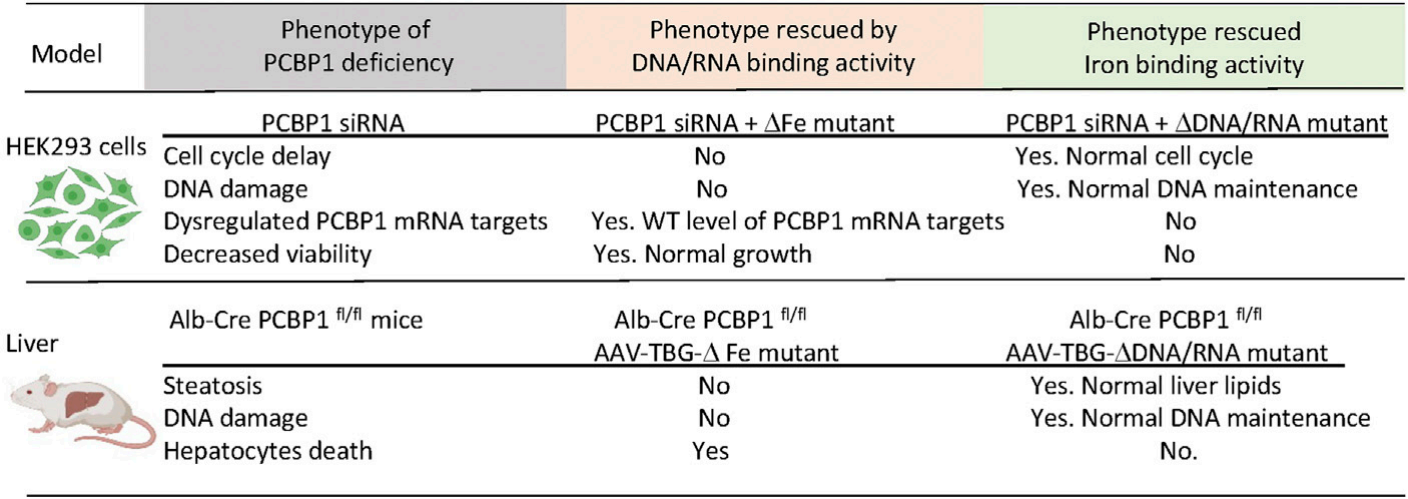
Dissection of the iron and DNA/RNA-binding activities of PCBP1. Loss of PCBP1 in cultured cells and livers results in a complex phenotype manifesting as DNA damage and reduced cell viability. PCBP1 protein contains three K homology (KH) domains. Mutations in iron-binding residues of KH1 (D82A), KH2 (E168A), and KH3 (E350A) block iron coordination by PCBP1. The ΔFe mutant with these substitutions cannot bind iron but preserves binding to DNA/RNA oligonucleotides. Mutations in nucleic acid-binding residues in KH1 (R40A), KH2 (R124A), KH3 (R306A) disrupt binding to single-stranded nucleic acids. With these substitutions, the ΔDNA/RNA mutant can bind iron but not DNA or RNA. HEK293 stable cell lines expressing PCBP1 variants were treated with siRNA targeting 3′UTR to deplete endogenous PCBP1. Mice with liver-specific deletion of PCBP1 were transduced with AAV expressing PCBP1 variants from the hepatocyte-specific TBG promoter. Expression of mutant variants of PCBP1 with the loss of iron- or DNA/RNA-binding activity complemented only part of the deletion phenotypes.

Liver-specific knockout of PCBP1 is a helpful model to express and study the multifunctional properties of PCBP1 *in vivo*. We introduced intact, wild type PCBP1 or variants with mutations in amino acid residues critical for the coordination of iron or oligonucleotides in each KH-domain of PCBP1 into adeno-associated virus serotype 8 (AAV) under the hepatocyte-specific promoter of thyroxine-binding globulin (TBG) (Patel et al., 2021). We then injected AAVs with PCBP1 variants into mice with PCBP1-deleted livers. The transduced variant protein demonstrated more expression in pericentral than in periportal areas of the liver, in agreement with previous studies (Bell et al., 2011). Despite spatial heterogenicity, the overall expression of transduced constructs in livers, as measured by immunoblotting, was the same or higher than endogenous protein. Transduction of the intact PCBP1 variant complemented the loss of endogenous PCBP1 in the liver and restored all examined parameters to the wild-type level. However, the transduction of mutant variants produced distinct phenotypes. Nucleic acid-binding activity was essential to prevent cell death. Transduction of the variant lacking RNA/DNA-binding but retaining iron binding activity in PCBP1-deleted livers was associated with persistence of elevated plasma ALT, indicating persistent liver damage and cell death. The iron-coordinating activity of PCBP1 was essential to prevent steatosis and DNA damage. Transduction with the variant lacking iron chaperone activity but retaining nucleic acid binding in PCBP1-deleted livers resulted in persistent steatosis and increased triglyceride accumulation. Surprisingly, the iron coordinating activity of PCBP1 was also essential in preventing DNA damage. PCBP1-depleted livers demonstrated increased TUNEL staining, a sign of DNA strand breaks and DNA damage. Livers expressing the RNA/DNA variant, which lacks iron binding, also exhibited higher TUNEL staining, indicating accumulation of damaged DNA. Thus, *in vitro* and *in vivo* approaches using HEK293 cells and mouse models, respectively, confirmed that iron chaperone activity is essential to prevent DNA damage. In contrast, the RNA/DNA-binding activity of PCBP1 is critical for viability (Patel et al., 2021) (Figure 4).

The essential nature of PCBP1 and PCBP2 proteins requires new approaches to studying the independent and overlapping functions of PCBP1 and PCBP2. AAV-mediated delivery of tissue-specific, codon-improved iCre-recombinase has advantages over transgenic mouse models with constitutive or inducible expression of conventional Cre-recombinase (Shimshek et al., 2002). For example, using AAV-mediated delivery of iCre in a ferroportin-floxed mouse strain using AAV9-Ple261(CLDN5)-iCre, ferroportin was depleted in the retina and brain vascular endothelial cells (Baumann et al., 2019). Transduction of AAV8-TBG-iCre in JMJD3 fl/fl mice produced successful depletion of the iron enzyme histone lysine demethylase in liver (Seok et al., 2018). The use of AAV-iCre also makes possible the simultaneous deletion of a floxed gene and the expression of its mutant variant. This approach was used to co-transduce iCre and wild-type or phosphorylation-resistant Wwtr1 mutant into Wwtr1 fl/fl livers (Wang et al., 2020). Simultaneous deletion of essential PCBP1 and PCBP2 during embryogenesis might be too detrimental for hepatocytes. The AAVs expressing iCre recombinase from liver-specific TBG promoters offer a unique opportunity for coordinated deletion of PCBP1, PCBP2, or both.

## High throughput approaches for identifying iron trafficking proteins

While animal models are very useful for studying iron chaperones in a physiological context of iron trafficking, other approaches are needed to identify new components of iron cofactor trafficking and delivery systems. Unbiased genome- and proteome-wide screens to explore protein interactions are powerful tools for the identification of new proteins participating in intracellular iron trafficking pathways. Three main approaches have been used: forward genetic screens, affinity purification, and proximity labeling. PCBP1 was identified as the iron chaperone for ferritin in a forward genetic screen conducted in baker’s yeast. In this approach, a human liver cDNA library was screened for genes that could bind and facilitate iron delivery to human ferritins when both were exogenously expressed in yeast (Shi et al., 2008). Other forward genetic screens based on the classical yeast two-hybrid assay have been used to identify human proteins interacting the iron transporters DMT1 and ferroportin 1. These screens identified PCBP2 as a chaperone involved in facilitating iron uptake and efflux in HEp-2 cells (Yanatori et al., 2014; Yanatori et al., 2016). Approaches based on the use of mass spectrometry for the identification of interacting proteins involved in iron trafficking have proven successful. In general, the protein of interest (the “*bait*”) is used to capture the interacting proteins (the “*prey*”), which are purified, identified, and quantified using spectrometric and proteomic analyses. The two main approaches used to capture those interacting proteins are affinity purification and proximity labeling, which sometimes are used in combination (Liu et al., 2020).

Affinity purifications are based on the isolation of protein complexes derived from live cells. The bait protein is pulled down from a cell lysate using specific antibodies and the interacting proteins are co-purified with the bait. All proteins are then identified *via* mass spectrometry. Although the endogenous bait protein may be immunoprecipitated using a very high affinity antibody against the bait, the most common approach is to use an epitope-tagged version of the bait protein instead, as this lessens the chance that the antibody will interfere with the native protein complexes. Because overexpression of the bait protein can lead to non-native interactions, we choose to use stable cell lines that inducibly express FLAG epitope-tagged bait proteins at moderate levels. We immunoprecipitate under native conditions using anti-FLAG-agarose and perform mass spectrometry identification of the affinity-purified interacting proteins. Cell lines that do not express the FLAG-tagged bait are used in parallel to identify non-specific interacting proteins. This approach allowed us to identify BolA2 as a PCBP1-interacting protein; subsequent studies determined that that PCBP1 and BolA2 form an iron chaperone complex for the assembly of cytosolic [2Fe–2S] clusters (Patel et al., 2019). A similar approach has been used to identify key factors involved in Fe-S cluster assembly and distribution in the cytoplasmic Fe-S biogenesis pathway (CIA) (Gari et al., 2012; Stehling et al., 2012; Maio et al., 2021). The coimmunoprecipitation-mass spectrometry approach allowed the identification of a [4Fe-4S] cluster targeting complex for nuclear proteins that contained MMS19, CIAO1, and FAM96B. These studies also identified the chromokinesin KIF4A as a MMS19-interactor and facilitated the characterization of KIF4A as [4Fe-4S] cluster binding protein (Ben-Shimon et al., 2018). More recently, Maio and colleagues described the SARS-CoV-2 RNA-dependent RNA polymerase, Nsp12, as an Fe-S cluster protein and used coimmunoprecipitation with mass spectrometry analyses to demonstrate the interaction of Nsp12 with components of the Fe-S biogenesis pathways as well as PCBP1 and BolA2 (Maio et al., 2021).

Although multiple studies have successfully used coimmunoprecipitation and mass spectrometry to identify binding partners in iron trafficking pathways, the transient and weak nature of these interactions may prevent the co-purification of protein complexes. Further challenges arise when the buffer conditions needed to solubilize, e.g., the nuclear compartment, prove too harsh to maintain interactions. Detection may be improved with the inclusion of crosslinking agents such as formaldehyde, which trap associations between binding partners during the purification steps. Additionally, a recently developed, targeted mass spectrometry approach provides better sensitivity and quantitation and is applicable when the interacting partners are known or suspected. This approach was used to confirm that the cytosolic Fe-S cluster assembly complex forms a metabolon consisting of scaffolding components, the targeting complex, and recipient apo-proteins (Fan et al., 2022).

Proximity labeling constitutes a powerful alternative to detect transient, less stable protein interactions in living cells, as this approach can identify proteins that are briefly close to the bait protein. The most common approach is proximity-dependent biotin identification (BioID), which is based on the expression of the bait protein fused with a promiscuous prokaryotic biotin ligase, BirA* (Choi-Rhee et al., 2004; Roux et al., 2012). BioID fusion proteins biotinylate adjacent proteins, within 10–20 nm from bait, then streptavidin resin selectively captures the biotinylated proteins, which are identified and quantified using mass spectrometry. BioID has its own intrinsic advantages and limitations. The two main advantages of BioID are the detection of both transient and stable protein-protein interactions in living cells and the insensitivity to non-native interactions that may occur during coimmunoprecipitation. The main drawbacks are interference of the BirA* moiety with natural interactions and false positives that may arise from close proximity to irrelevant proteins (Roux et al., 2018). The simultaneous use of both coimmunoprecipitation and BioID approaches is complementary and can be achieved by using a single stable cell line construct and a multi-tag workflow (Liu et al., 2020).

All protein-protein interactions identified *via* proteomic analysis must be validated by other methodologies. These may include reciprocal coimmunoprecipitation or streptavidin pull-downs followed by immunoblotting (Frey et al., 2016). Protein pairs in close proximity (<40 nm) may be detected *in situ* by the use of proximity ligation assay (PLA). This method employs pairs of PLA probes, which are specific antibodies conjugated to complimentary DNA oligonucleotides. If the two tested proteins are close, PLA pairs can hybridize to make circular DNA, which can be amplified and fluorescently labeled for visualization by fluorescence microscopy (Alam, 2018).

### Global cysteine reactivity to identify Fe-S cluster coordination

The identification of PCBP1-BolA2 as an iron chaperone complex for [2Fe-2S] cluster assembly on Glrx3 - BolA2 prompts us to further investigate the roles of iron chaperone complexes in Fe-S assembly. While protein-interactome studies are a valuable approach, a high-throughput tool to examine global Fe-S cluster coordination would powerfully reveal the breadth of specific iron delivery systems. The systematic identification and characterization of Fe/Fe-S cluster binding sites in proteins has been hindered due to the absence of canonical sequence motifs defining Fe/Fe-S cluster binding sites and the highly reactive nature of Fe-S clusters that makes them prone to degradation (Rouault, 2015; Maio and Rouault, 2016). Conventional biophysical and spectroscopic approaches, widely used for the characterization of Fe-S cluster proteins, require purified protein (Lindahl and Holmes-Hampton, 2011; Dlouhy and Outten, 2013; Pandelia et al., 2015), which complicates the study of these proteins on a global scale. However, recent advances in chemo-proteomics methodologies have increased our ability to predict, identify and characterize Fe-S cluster proteins.

**FIGURE 5:**
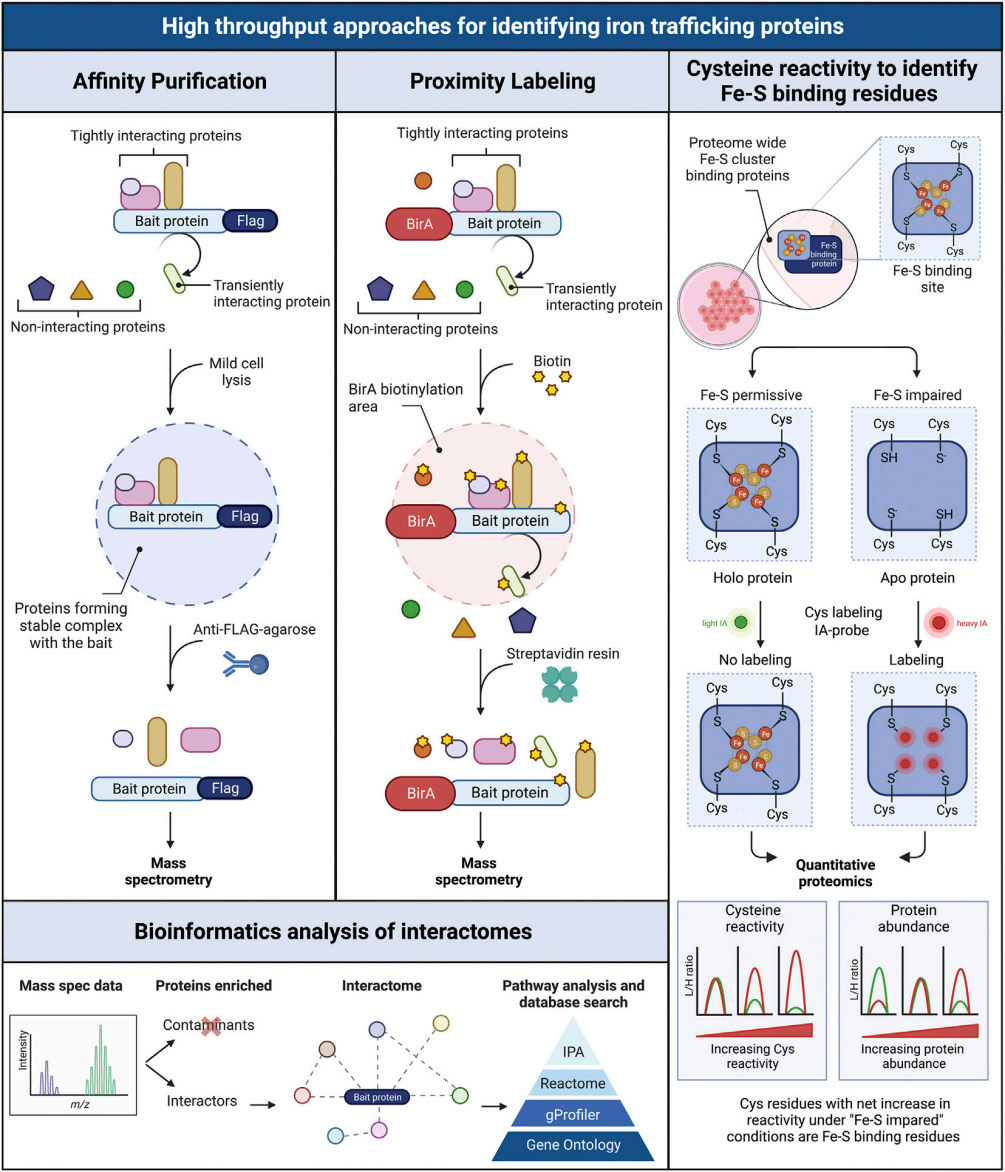
Schematic overview of three high throughput workflows for screening interacting intracellular iron trafficking proteins. Affinity purification (left panel): the flag tagged bait protein is pulled down from cell lysates (mild conditions) using an anti-Flag agarose resin. Only tightly interacting proteins forming stable complexes with the bait are coimmunoprecipitated. Interacting proteins are identified *via* mass spectrometry. Proximity labeling BioID (central panel): the bait protein is expressed fused to the biotin ligase BirA, which biotinylates proteins located in close proximity to the bait, including tightly and transiently interacting proteins. Biotinylated proteins are purified using streptavidin resin and identified *via* mass spectrometry. Bioinformatics analysis of interactomes (bottom left panel): the obtained proteomic data are filtered to separate non-specific interactors and contaminants from true interactors. Interactomes are then visualized in networks and analyzed for enriched canonical pathways related to iron trafficking and metabolism. Cysteine reactivity to identify Fe-S binding residues in proteomes (right panel): Cysteines in Fe-S cluster binding sites of proteins are analyzed proteome-wide under Fe-S permissive (control conditions that favor the formation of Fe-S clusters) and Fe-S impaired conditions (i.e. treatment with iron chelator or perturbation of Fe-S biogenesis pathway to impair Fe-S cluster formation). The existence of *holo* or *apo* forms of the Fe-S proteins is tested by isotopically labeling free cysteines using light (L) and heavy (H) iodoacetamide (IA), one per condition. The presence of the cluster in *holo* proteins protects the cysteines from IA-alkylation (*No labeling*). In turn, the Fe-S binding cysteines in *apo* proteins are available for IA-alkylation (*Labeling*). The L/H ratio is determined *via* quantitative proteomics to measure the reactivity of the cysteines in the Fe-S proteins. A parallel proteomic quantification accounts for any change in protein abundance between treatments. Net increases in cysteine reactivity under “Fe-S” impaired conditions compared to control, are indicative of the loss of Fe-S clusters. Figured adapted from (96).

Quantitative cysteine reactivity profiling is a recently developed mass spectrometry-based strategy to assess Fe-S clusters occupancy in native proteomes. It uses quantitative chemo-proteomics to assess the presence of intact clusters in complex biological systems (Bak and Weerapana, 2023). Fe-S binding proteins use cysteine residues to coordinate clusters (Andreini et al., 2018). This method uses the inherent reactivity of cysteine residues to determine if a given cysteine residue in a protein is binding an Fe-S cluster by isotopically labeling the thiol groups on cysteine residues (Weerapana et al., 2010). The presence of the cluster protects the cysteine from chemical modification (i.e., alkylation), while the loss of the Fe-S cluster will increase the reactivity of the cysteines from the cluster binding site. The change in cysteine reactivity is measured using the isoTOP-ABPP approach, which is based on the labeling of reactive cysteines with isotopically tagged variants of the iodoacetamide-alkyne (IA-alkyne) probe (Abo et al., 2018). To account for changes in protein levels between treatment conditions, protein abundance is measured in parallel *via* reductive isotope dimethyl labeling (ReDiMe) and quantitative mass spectrometry analysis (Boersema et al., 2009; Bak and Weerapana, 2023). In this manner, conditions that destabilize native cluster coordination (depletion of cellular iron, disruption of cluster assembly machinery) may be compared to conditions that support cluster assembly. Although this method was initially developed for use in the less complex proteome of *E. coli*, it could be adapted for use in the complex proteomes of mammals. Strategies such as organelle enrichment, subcellular fractionation, and interactome purification could reduce complexity and improve the detection of Fe-S cluster proteins in the human proteome. These global approaches to study protein interactomes and iron cofactor occupancy are summarized in Figure 5.

### Bioinformatics approaches tailored for the analysis of iron metabolism

Global, high-throughput analyses require the application of computational and bioinformatic approaches for the handling and analysis of large datasets. Frequently, the discovery of new proteins or pathways is impeded by the lack of bioinformatics tools and databases tailored for the analysis of iron metabolism-related elements. Once proteomic data are generated and the true positives separated from the false positives, workflows specifically tailored to inspect iron-metabolism-related elements are performed on the entire interactome. This requires the existence of curated databases containing, for example, updated catalogues of annotated Fe or Fe-S proteins in the human proteome, cofactor assembly and delivery pathways, and metabolic pathways in which Fe and Fe-S cluster proteins participate. However, these resources are scarce and incomplete. Only recently (2021–2022) have iron-related canonical pathways been added to the curated database from QIAGEN Ingenuity Pathway Analysis (IPA, Qiagen) (https://www.qiagen.com/ingenuity). This database includes pathways related with iron deficiency, iron homeostasis, iron transport, iron overload, ferroptosis, and the transcription factor NRF2. These canonical pathways have facilitated the analysis of large transcriptomic datasets, for example, exploring the effects of bariatric surgery on the activation of iron absorption pathways (Evers et al., 2022) or modeling macrophage iron sequestration during host defense against Aspergillus (Adhikari et al., 2022). However, improving the availability of curated databases focused on iron trafficking and metabolism will potentiate our capacities for prediction and analysis of high-throughput datasets.

## Approaches for other nutrient metals

While the biophysical and biochemical characteristics of other nutrient metal cofactors differ from those of iron cofactors, the transient nature of the interactions between metallochaperones and client proteins is also characteristic of other metal distribution systems, making the methods described here suitable for the analysis of other metal trafficking systems. For example, AAV transduction of murine models of metallochaperone deficiency is a versatile approach and has been used to study truncated forms of ATP7B in a mouse model of Wilson disease. Fluorescent metal sensors have been developed for Cu, Zn, Ca, Mg, etc., which are commercially available. For example, some protein interactors of copper chaperones, transporters and regulators have been uncovered by conventional yeast two-hybrid analysis (van Dongen et al., 2004) (Ohrvik and Wittung-Stafshede, 2015) (Lim et al., 2006a) (Lim et al., 2006b) (Larin et al., 1999) and coimmunoprecipitation followed by mass spectrometry analysis (Matson Dzebo et al., 2018) (Comstra et al., 2017). Examples of the use of BioID coupled to whole proteome analysis for the study of protein interactomes in the context of metal trafficking are scarce. The few examples include the use of BioID constructs to explore some protein interactors of the copper chaperone for superoxide dismutase (CCS) followed by detection *via* western blot without mass spectrometry (Grasso et al., 2021), and the interactome of the copper chaperone SCO1 determined during the description of a high-throughput BioID technique (Liu et al., 2018). Therefore, the use of the unbiased BioID approach described here for the study of the protein-protein interactions that drive the flux of metals inside cells is promising and remain to be explored. In addition to Fe-S clusters, cysteine residues coordinate other metals such as copper (I) and zinc (II) (Arnesano et al., 2002). Just as Fe-S clusters protect coordinating cysteine residues from alkylation (Bak and Weerapana, 2023), copper (I) and zinc (II) binding also attenuate the reactivity of the coordinating cysteine thiolates (Apuy et al., 2001; Glauninger et al., 2018; Novoa-Aponte et al., 2019). Hence, the cysteine reactivity assay seems feasible for the whole proteome analysis of *apo* and *holo* states of proteins in the context of copper and zinc trafficking, although this has yet to be demonstrated.
